# Performance Optimizations with Single-, Bi-, Tri-, and Quadru-Objective for Irreversible Diesel Cycle

**DOI:** 10.3390/e23070826

**Published:** 2021-06-28

**Authors:** Shuangshuang Shi, Lingen Chen, Yanlin Ge, Huijun Feng

**Affiliations:** 1 Institute of Thermal Science and Power Engineering, Wuhan Institute of Technology, Wuhan 430205, China; shishuangshuang20@163.com (S.S.); huijunfeng@139.com (H.F.); 2 School of Mechanical & Electrical Engineering, Wuhan Institute of Technology, Wuhan 430205, China

**Keywords:** irreversible Diesel cycle, power output, thermal efficiency, ecological function, power density, finite time thermodynamics

## Abstract

Applying finite time thermodynamics theory and the non-dominated sorting genetic algorithm-II (NSGA-II), thermodynamic analysis and multi-objective optimization of an irreversible Diesel cycle are performed. Through numerical calculations, the impact of the cycle temperature ratio on the power density of the cycle is analyzed. The characteristic relationships among the cycle power density versus the compression ratio and thermal efficiency are obtained with three different loss issues. The thermal efficiency, the maximum specific volume (the size of the total volume of the cylinder), and the maximum pressure ratio are compared under the maximum power output and the maximum power density criteria. Using NSGA-II, single-, bi-, tri-, and quadru-objective optimizations are performed for an irreversible Diesel cycle by introducing dimensionless power output, thermal efficiency, dimensionless ecological function, and dimensionless power density as objectives, respectively. The optimal design plan is obtained by using three solution methods, that is, the linear programming technique for multidimensional analysis of preference (LINMAP), the technique for order preferences by similarity to ideal solution (TOPSIS), and Shannon entropy, to compare the results under different objective function combinations. The comparison results indicate that the deviation index of multi-objective optimization is small. When taking the dimensionless power output, dimensionless ecological function, and dimensionless power density as the objective function to perform tri-objective optimization, the LINMAP solution is used to obtain the minimum deviation index. The deviation index at this time is 0.1333, and the design scheme is closer to the ideal scheme.

## 1. Introduction

As a further extension of traditional irreversible process thermodynamics, finite time thermodynamics [1,2,3,4,5,6,7,8,9,10,11,12,13] have been applied to analyze and optimize performances of actual thermodynamic cycles, and great progress has been made. The application of finite time thermodynamics to study the optimal performance of Diesel cycles represents a new technology for improving and optimizing Diesel heat engines, and a new method for studying Diesel cycles has been developed. Assuming the working fluid’s specific heats are constants [14,15,16,17,18,19,20,21,22,23,24] and vary with its temperature [25,26,27,28,29,30,31,32], many scholars have studied the performance of irreversible Diesel cycles with various objective functions, such as power output (P), thermal efficiency (η), and ecological functions (E, which was defined as the difference between the exergy flow rate and the exergy loss).

In addition to the above objective functions, Sahin et al. [33,34] took power density ($P_{d}$, defined as the ratio of the cycle P to the maximum specific volume) as a new optimization criterion to optimize Joule–Brayton engines and found that the heat engine designed under the $P_{d}$ criterion has higher η and a smaller size when no loss is considered. Chen et al. [35] introduced the objective function $P_{d}$ into the thermodynamic analysis and optimization of the Atkinson cycle. Atmaca and Gumus [36] compared and analyzed the optimal performance of a reversible Diesel cycle based on the P, $P_{d}$, and effective P (which was defined as the product of power output and thermal efficiency) criteria. Raman and Kumar [37] conducted thermodynamic analysis and optimization of a reversible Diesel cycle under the criteria of P, $P_{d}$, and effective P when the working fluid’s specific heats were linearly functioning with temperature. Rai and Sahoo [38] analyzed the influences of different losses on the effective P, effective $P_{d}$, and total heat loss of an irreversible Diesel cycle when the working fluid’s specific heats were non-linearly functioning with temperature. Gonca and Palaci [39] analyzed and compared design parameters under the effective P and effective $P_{d}$ criteria of an irreversible Diesel cycle.

The research mentioned above only optimized a single-objective function and did not optimize multiple objective functions at the same time. Therefore, NSGA-II can be used to solve a multi-objective optimization (MOO) problem, and MOO can be performed for the combination of different objective functions.

Ahmadi et al. [40,41,42,43] carried out MOO for an irreversible radiant heat engine [40], fuel cell combined cycle [41,42], and Lenoir heat engine [43] with different objective functions. Shi et al. [44] and Ahmadi et al. [45] performed MOO of the Atkinson cycle when the working fluid’s specific heats were constants [44] and varied with temperature non-linearly [45]. Gonzalez et al. [46] performed MOO on P, η, and entropy generation of an endoreversible Carnot engine and analyzed the stability of the Pareto frontier. Ata et al. [47] performed parameter optimization and sensitivity analysis for an organic Rankine cycle with a variable temperature heat source. Herrera et al. [48] and Li et al. [49] performed MOO of η and emissions of a regenerative organic Rankine cycle. Garmejani et al. [50] performed MOO of P, exergy efficiency, and investment cost for a thermoelectric power generation system. Tang et al. [51] and Nemogne et al. [52] performed MOO of an irreversible Brayton cycle [51] and an absorption heat pump cycle [52]. MOO has been applied for performance optimization of various processes and cycles [53,54,55,56].

Reference [24] established a relatively complete irreversible Diesel cycle model and studied the optimal performance of E. Firstly, based on the model established in the reference [24], this paper studies the optimal $P_{d}$ performance of an irreversible Diesel cycle while considering the impacts of the cycle temperature ratio and three loss issues. Secondly, the maximum specific volume, maximum pressure ratio, and η are compared under the maximum P and maximum $P_{d}$ criteria. Thirdly, applying NSGA-II with a compression ratio as the decision variable and cycle dimensionless P ($\bar{P}$, which is defined as P divided by maximum P), η, dimensionless $P_{d}$ (${\bar{P}}_{d}$, which is defined as $P_{d}$ divided by maximum $P_{d}$), and dimensionless E ($\bar{E}$, which is defined as E divided by maximum E) as objective functions, the single-, bi-, tri-, and quadru-objective optimizations of an irreversible Diesel cycle are performed. Through three different solutions, that is, LINMAP, TOPSIS, and Shannon entropy, the deviation indexes obtained under different solutions are compared, and the optimized design scheme with the smallest deviation index is finally obtained.

## 2. Cycle Model

**Figure 1:**
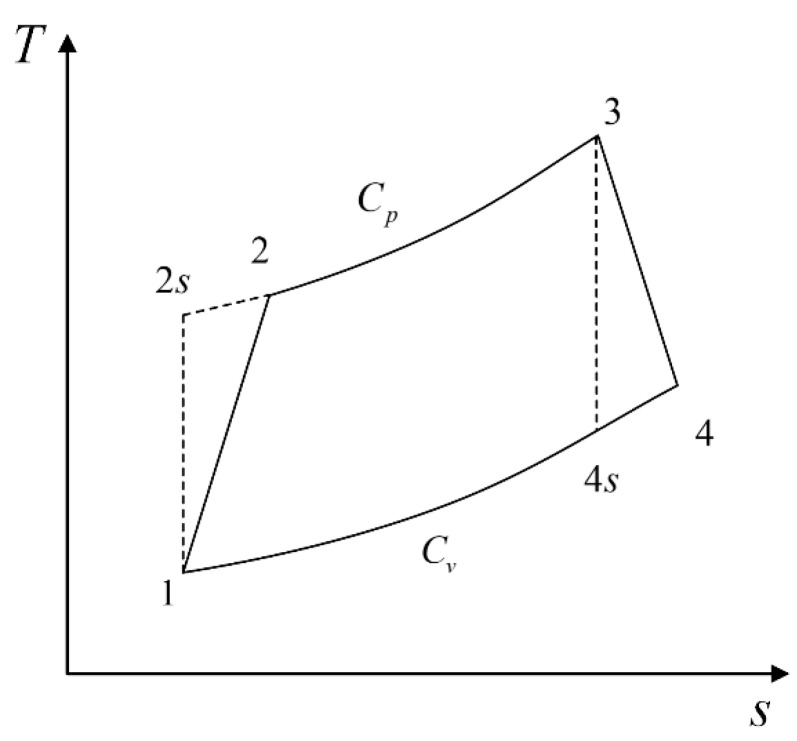
T−s representation of the Diesel cycle.

**Figure 2:**
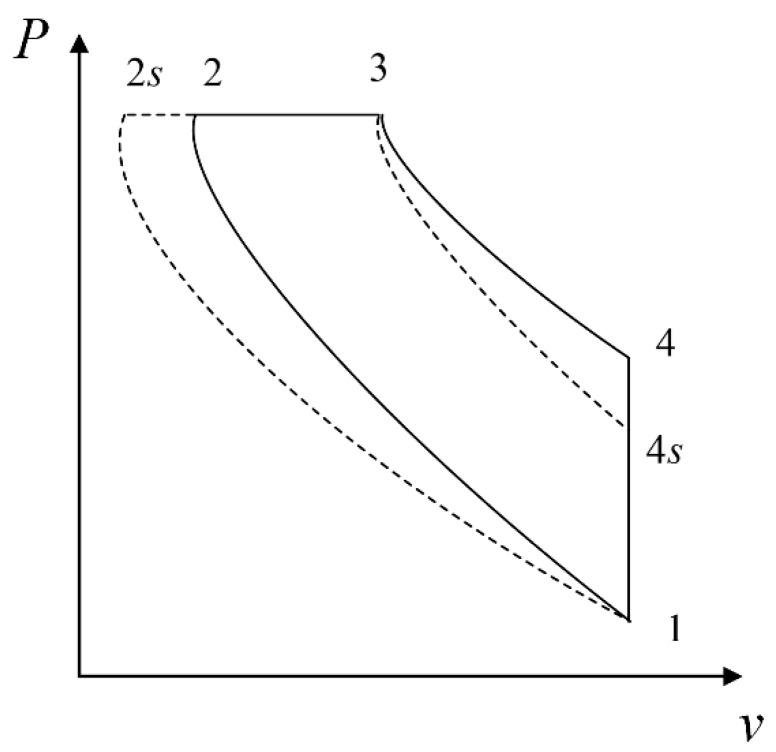
P−v representation of the Diesel cycle.

The working fluid is assumed to be an ideal gas. Figure 1 and Figure 2 show the T−s and P−v diagrams of an irreversible Diesel cycle. It can be seen that 1−2 is an adiabatic process, 2−3 is a constant-pressure process, 3−4 is an adiabatic process, and 4−1 is a constant-volume process. The processes 1−2s and 3−4s are the isentropic and adiabatic processes, respectively.

The heat absorption and release rates are, respectively,

(1) $${\dot{Q}}_{in}=\dot{m}C_{p}(T_{3}-T_{2})$$

(2) $${\dot{Q}}_{out}=\dot{m}C_{v}(T_{4}-T_{1})$$

where $\dot{m}$ is the mass flow rate, and $C_{v}$ and $C_{p}$ are the specific heats under constant volume and pressure, respectively.

Some internal irreversibility loss (IIL) is caused by friction, turbulence, and viscous stress. The irreversible compression and expansion internal efficiencies are expressed as [16,19,20,30]

(3) $$\eta_{c}=\left(T_{2s}-T_{1}\right)/\left(T_{2}-T_{1}\right)$$

(4) $$\eta_{e}=\left(T_{3}-T_{4}\right)/\left(T_{3}-T_{4s}\right)$$

The cycle compression ratio γ and temperature ratio τ are

(5) $$\gamma =V_{1}/V_{2}$$

(6) $$\tau =T_{3}/T_{1}$$

According to the property of isentropic process, one has

(7) $$T_{2s}=T_{1}\gamma^{k-1}$$

(8) $${\left(T_{3}/T_{2s}\right)}^{k}=T_{4s}/T_{1}$$

According to Equations (3)–(8), one has

(9) $$T_{2}=T_{1}[(\gamma^{k-1}-1)/\eta_{c}+1]$$

(10) $$T_{4s}=\tau^{k}T_{1}/\gamma^{k(k-1)}$$

(11) $$T_{4}=T_{1}[\tau^{k}\eta_{e}/\gamma^{k(k-1)}-\tau \eta_{e}+\tau ]$$

For the actual heat engine, there is heat transfer loss (HTL) between the working fluid and the cylinder. According to Refs. [14,24,27], it is known that the fuel exothermic rate is equal to the sum of the total endothermic rate and the HTL rate; one has

(12) $${\dot{Q}}_{leak}=A-{\dot{Q}}_{in}=B(T_{3}+T_{2}-2T_{0})$$

where A is the fuel exothermic rate and B is the HTL coefficient.

Similarly, as the piston generates friction with the cylinder wall when running at high speed, the friction loss (FL) of the cycle cannot be ignored. As a four-stroke heat engine, a Diesel heat engine has four strokes of intake, compression, expansion, and exhaust, and all of them produce FL. According to Refs. [24,32], for the treatment of FL in each stroke, the FL during compression and expansion is included in internal irreversible losses. According to Refs. [57,58,59], the piston motion resistance in the intake process is greater than that in the exhaust process. If the friction coefficient in the exhaust process is μ, the equivalent friction coefficient, which includes the pressure drop loss in the intake process, is 3μ. The friction coefficients on the exhaust and intake stroke are μ and 3μ, respectively. There is a linear relationship between friction force and speed: *fμ* = *−μv* = *−μdx/dt*, where x is the piston displacement and μ is the FL coefficient. The power consumed due to FL during the exhaust and intake strokes can be derived as

(13) $$P_{\mu }=dW_{\mu }/dt=4\mu {\left(dx/dt\right)}^{2}=4\mu v^{2}$$

For a Diesel cycle, the average speed of the piston in four reciprocating motions is

(14) $$\bar{v}=4Ln$$

where n is the rotating speed and L is the stroke length.

Therefore, the power consumed by cycle FL is

(15) $$P_{\mu }=4\mu {\left(4Ln\right)}^{2}=64\mu {\left(Ln\right)}^{2}$$

The cycle P and η are, respectively,

(16) $$P={\dot{Q}}_{in}-{\dot{Q}}_{out}-P_{\mu }=\dot{m}[C_{p}(T_{3}-T_{2})-C_{v}(T_{4}-T_{1})]-64\mu {\left(Ln\right)}^{2}$$

(17) $$\eta =\frac{P}{{\dot{Q}}_{in}+{\dot{Q}}_{leak}}=\frac{\dot{m}[C_{p}(T_{3}-T_{2})-C_{v}(T_{4}-T_{1})]-64\mu {\left(Ln\right)}^{2}}{\dot{m}C_{p}(T_{3}-T_{2})+B(T_{2}+T_{3}-2T_{0})}$$

According to the definition of *P_d_* in Refs. [33,34,35], the $P_{d}$ is expressed as

(18) $$P_{d}=P/v_{4}$$

According to Refs. [38,39], the total volume *v_t_*, stroke volume *v_s_*, and gap volume $v_{c}$ of the cycle are defined as

(19) $$v_{t}=v_{s}+v_{c}$$

(20) $$v_{s}=\pi d^{2}L/4$$

(21) $$v_{c}=\pi d^{2}L/4(\gamma -1)$$

In the Diesel cycle, $v_{t}=v_{max}=v_{1}$, $v_{c}=v_{2}$. According to Equations (5) and (17)–(19), one has

(22) $$P_{d}=P/v_{max}=P/v_{t}=4(\gamma -1)P/\pi d^{2}L\gamma$$

According to Ref. [24], an irreversible Diesel cycle has four kinds of entropy generation due to FL, HTL, IIL, and exhaust stroke to the environment. The four entropy generation rates are expressed as

(23) $$\sigma_{q}=B[1/T_{0}-2/(T_{2}+T_{3})](T_{3}+T_{2}-2T_{0})$$

(24) $$\sigma_{\mu }=P_{\mu }/T_{0}=64\mu {\left(Ln\right)}^{2}/T_{0}$$

(25) $$\sigma_{2s\to 2}=\dot{m}\int_{T_{2s}}^{T_{2}}C_{p}dT/T=\dot{m}C_{p}\ln(T_{2}/T_{2s})$$

(26) $$\sigma_{4s\to 4}=\dot{m}\int_{T_{4s}}^{T_{4}}C_{v}dT/T=\dot{m}C_{v}\ln(T_{4}/T_{4s})$$

(27) $$\sigma_{pq}=\dot{m}\int_{T_{1}}^{T_{4}}C_{v}dT(1/T_{0}-1/T)=\dot{m}C_{v}[(T_{4}-T_{1})/T_{0}+\ln(T_{1}/T_{4})]$$

Therefore, the total entropy generation rate is

(28) $$\sigma =\sigma_{q}+\sigma_{\mu }+\sigma_{2s\to 2}+\sigma_{4s\to 4}+\sigma_{pq}$$

According to the definition of E in Ref. [24], the E is expressed as

(29) $$E=P-T_{0}\sigma$$

According to the processing method of Refs. [35,44], $\bar{P}$, ${\bar{P}}_{d}$, and $\bar{E}$ are respectively defined as

(30) $$\bar{P}=P/P_{\max}$$

(31) $${\bar{P}}_{d}=P_{d}/{\left(P_{d}\right)}_{\max}$$

(32) $$\bar{E}=E/E_{\max}$$

According to Equations (4), (9) and (11) and given the compression ratio *γ*, the initial cycle temperature $T_{1}$, and the cycle temperature ratio τ, by solving the temperatures at the 2, 3, and 4 state points, the corresponding numerical solutions of $\bar{P}$, η, ${\bar{P}}_{d}$, and $\bar{E}$ can be obtained.

## 3. Maximum Power Density Optimization

The working fluid is assumed to be an ideal gas. According to the nature of the air, $T_{0}=300\;K$, $T_{1}=350\;K$, $\dot{m}=1\;mol/s$, k=1.4, $C_{v}=20.78\;J/(mol\cdot K)$, and τ=5.78−6.78. According to Refs. [24,44], the cycle parameters are determined: γ=1−100, B=2.2 W/K, μ=1.2 kg/s, L=0.07 m and $n=30\;s^{-1}$.

**Figure 3:**
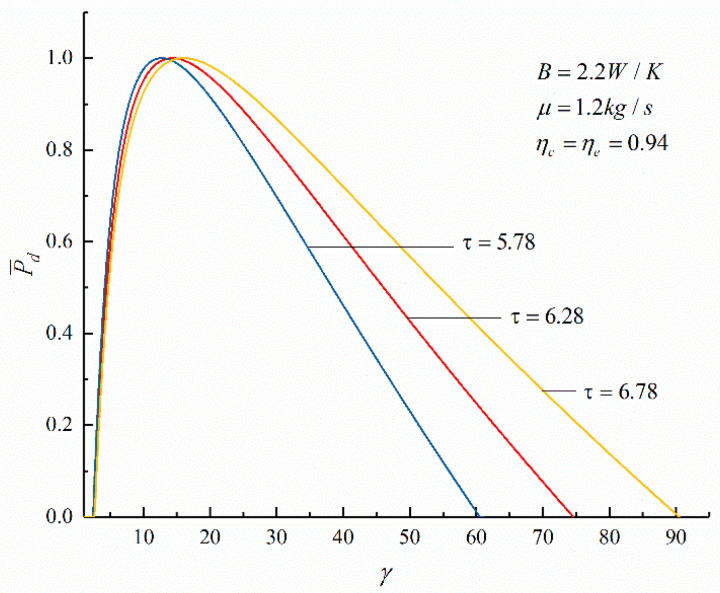
The effect of τ on ${\bar{P}}_{d}-\gamma$.

**Figure 4:**
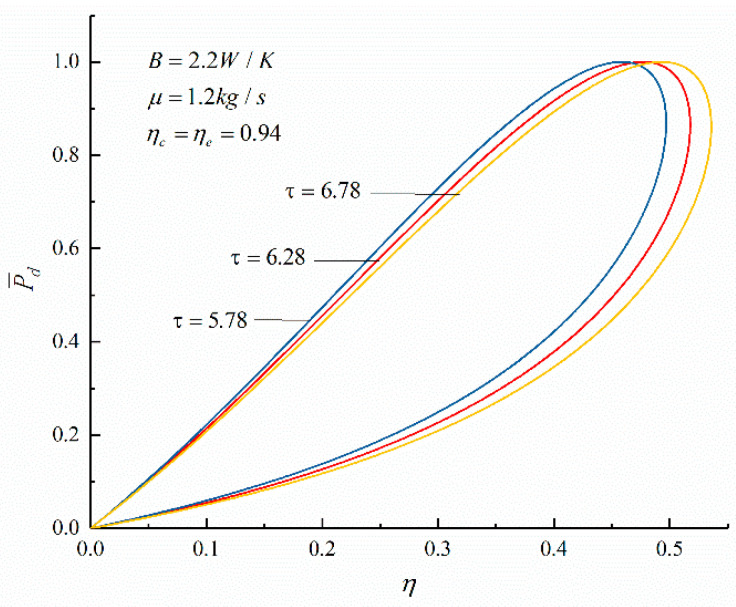
The effect of τ on ${\bar{P}}_{d}-\eta$.

**Figure 5:**
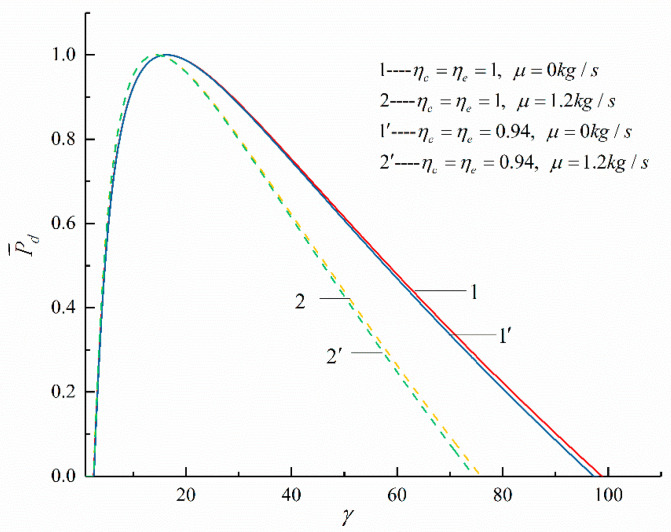
The effects of $\eta_{c}$, $\eta_{e}$, B, and b on ${\bar{P}}_{d}-\gamma$.

**Figure 6:**
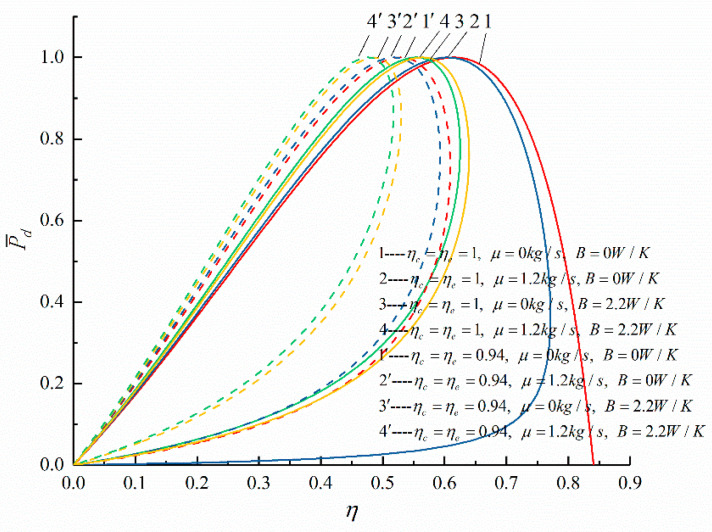
The effects of $\eta_{c}$, $\eta_{e}$, and b on ${\bar{P}}_{d}-\eta$.

The relationships between the objective functions (${\bar{P}}_{d}$ and η) of an irreversible Diesel cycle and the cycle design parameters (the cycle temperature ratio, HTL, FL, and IIL) are shown in Figure 3, Figure 4, Figure 5 and Figure 6. It can be noticed that the relationship between ${\bar{P}}_{d}$ and γ (${\bar{P}}_{d}-\gamma$) is a parabolic-like one. When no loss is considered, the relationship between ${\bar{P}}_{d}$ and η (${\bar{P}}_{d}-\eta$) is a parabolic-like one, and when there is loss, the relationship curve of ${\bar{P}}_{d}-\eta$ is a loop-shaped one.

Figure 3 and Figure 4 show the effects of τ on the performances of ${\bar{P}}_{d}-\gamma$ and ${\bar{P}}_{d}-\eta$. According to Figure 3, it can be seen that there is an optimal compression ratio ($\gamma_{{\bar{P}}_{d}}$), which makes ${\bar{P}}_{d}$ reach the maximum. As τ increases, $\gamma_{{\bar{P}}_{d}}$ increases; when τ increases from 5.78 to 6.78, $\gamma_{{\bar{P}}_{d}}$ increases from 12.7 to 16 (an increase of 25.98%). According to Figure 4, there is thermal efficiency ($\eta_{{\bar{P}}_{d}}$) corresponding to the maximum ${\bar{P}}_{d}$. As τ increases, $\eta_{{\bar{P}}_{d}}$ increases; when τ increases from 5.78 to 6.78, $\eta_{{\bar{P}}_{d}}$ increases from 45.82% to 49.29% (an increase of 7.40%). It can be seen that with the increase in τ, $\gamma_{{\bar{P}}_{d}}$, and $\eta_{{\bar{P}}_{d}}$ corresponding to the maximum ${\bar{P}}_{d}$ also increases.

Figure 5 and Figure 6 show the ${\bar{P}}_{d}-\gamma$ and ${\bar{P}}_{d}-\eta$ curves of the cycle when there are three different losses. Table 1 lists $\eta_{{\bar{P}}_{d}}$ when considering different losses and the percentage of the decrease in $\eta_{{\bar{P}}_{d}}$ compared with when no loss is considered. It can be seen that, with the increase in the losses considered, $\eta_{{\bar{P}}_{d}}$ decreases. When the three losses are considered at the same time, $\eta_{{\bar{P}}_{d}}$ decreases by 22.55% compared to that without any losses. According to Figure 5, it can be seen that as the compression ratio increases, ${\bar{P}}_{d}$ first increases and then decreases. According to Figure 6, it can be seen that when there are increases in HFL, FL, and IIL, $\eta_{{\bar{P}}_{d}}$ corresponding to the maximum ${\bar{P}}_{d}$ decreases.

**Figure 7:**
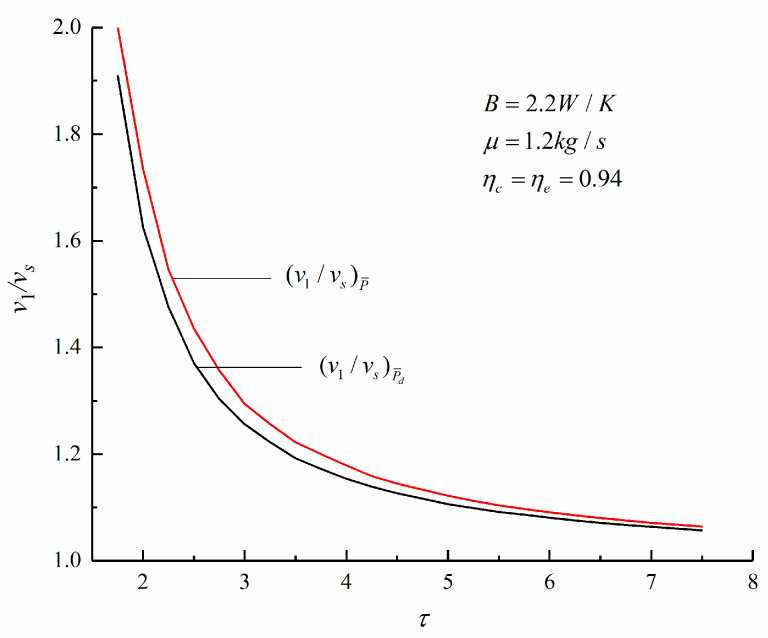
Variations of various $v_{1}/v_{s}$ with τ.

**Figure 8:**
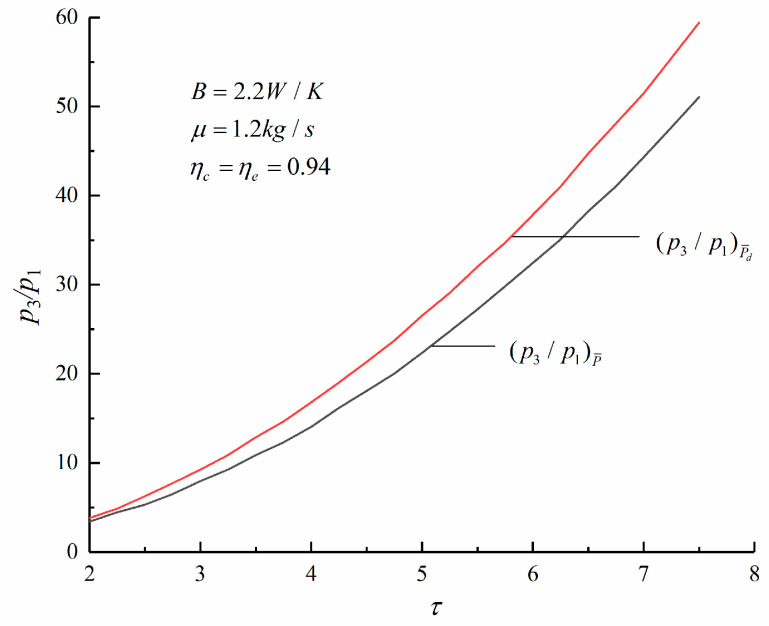
Variations of various $p_{3}/p_{1}$ with τ.

**Figure 9:**
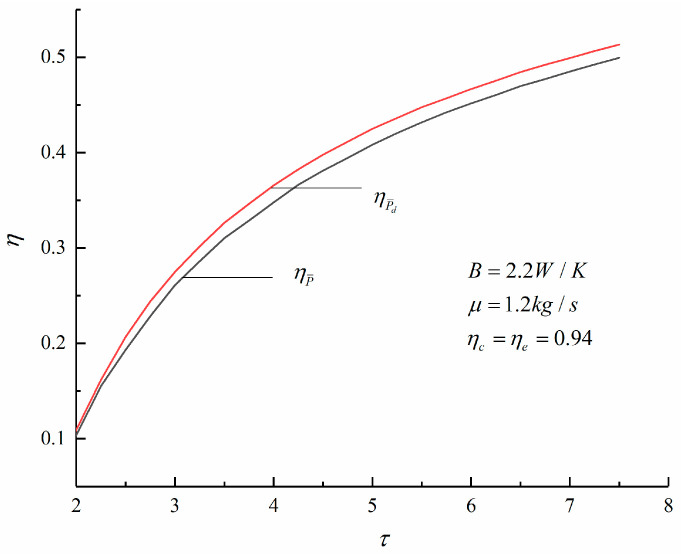
Variations of various η with τ.

Figure 7, Figure 8 and Figure 9 show the change trends of the corresponding maximum specific volume, maximum pressure ratio, and *η* with the τ under the maximum $\bar{P}$ and maximum ${\bar{P}}_{d}$ criteria of an irreversible Diesel cycle. According to Figure 7 and Figure 8, compared with the corresponding results under the maximum $\bar{P}$ criterion, the maximum specific volume is smaller and the maximum pressure ratio is larger under the maximum ${\bar{P}}_{d}$ criterion. It is observed that the Diesel heat engine designed under the maximum ${\bar{P}}_{d}$ criterion has a smaller size.

According to Figure 9, the η of the cycle under the maximum ${\bar{P}}_{d}$ criterion is higher. When τ=6.28, the η obtained under the maximum $\bar{P}$ and maximum ${\bar{P}}_{d}$ criterion are 46.04% and 47.64%, respectively. The latter is an increase of 3.54% over the former. Therefore, compared with the maximum $\bar{P}$ criterion, the engine designed under the maximum ${\bar{P}}_{d}$ criterion has a smaller size and a higher η.

## 4. Multi-Objective Optimization with Power Output, Thermal Efficiency, Ecological Function, and Power Density

MOO cannot make multiple objective functions reach the optimal value at the same time. The best compromise is achieved by comparing the pros and cons of each objective function. Therefore, the MOO solution set is not unique, and a series of feasible alternatives can be obtained, which are called Pareto frontiers. In this section, $\bar{P}$, η, $\bar{E}$, and ${\bar{P}}_{d}$ are used as objective functions; the compression ratio (γ) is used as an optimization variable; and NSGA-II [44,45,46,47,48,49,50,51,52] is used to perform bi-, tri-, and quadru-objective optimizations for an irreversible Diesel cycle. Through three different solutions, that is, LINMAP, TOPSIS, and Shannon entropy, the optimization results under different objective function combinations are obtained.

In the LINMAP solution, a minimum spatial distance from the ideal point is selected as the desired final optimal solution. In the TOPSIS solution, a maximum distance from the non-ideal point and a minimum distance from the ideal point are selected as the desired final optimal solution. In the Shannon entropy solution, a maximum value corresponding to a certain objective function is selected as the desired final optimal solution.

The optimization problems are solved with different optimization objective combinations, which form different MOO problems.

The six bi-objective optimization problems are as follows:

(33) $$\max\left\{ \begin{array}{l} \bar{P}(\gamma )\\ \eta (\gamma ) \end{array}\right.,\max\left\{ \begin{array}{l} \bar{P}(\gamma )\\ \bar{E}(\gamma ) \end{array}\right.,\max\left\{ \begin{array}{l} \bar{P}(\gamma )\\ {\bar{P}}_{d}(\gamma ) \end{array}\right.,\max\left\{ \begin{array}{l} \eta (\gamma )\\ \bar{E}(\gamma ) \end{array}\right.,\max\left\{ \begin{array}{l} \eta (\gamma )\\ {\bar{P}}_{d}(\gamma ) \end{array}\right.,\max\left\{ \begin{array}{l} \bar{E}(\gamma )\\ {\bar{P}}_{d}(\gamma ) \end{array}\right.$$

The four tri-objective optimization problems are as follows:

(34) $$\max\left\{ \begin{array}{l} \bar{P}(\gamma )\\ \eta (\gamma )\\ \bar{E}(\gamma ) \end{array}\right.,\max\left\{ \begin{array}{l} \bar{P}(\gamma )\\ \eta (\gamma )\\ {\bar{P}}_{d}(\gamma ) \end{array}\right.,\max\left\{ \begin{array}{l} \bar{P}(\gamma )\\ \bar{E}(\gamma )\\ {\bar{P}}_{d}(\gamma ) \end{array}\right.,\max\left\{ \begin{array}{l} \eta (\gamma )\\ \bar{E}(\gamma )\\ {\bar{P}}_{d}(\gamma ) \end{array}\right.$$

The one quadru-objective optimization problem is as follows:

(35) $$\max\left\{ \begin{array}{l} {\bar{P}}_{d}(\gamma )\\ \eta (\gamma )\\ \bar{E}(\gamma )\\ {\bar{P}}_{d}(\gamma ) \end{array}\right.$$

**Figure 10:**
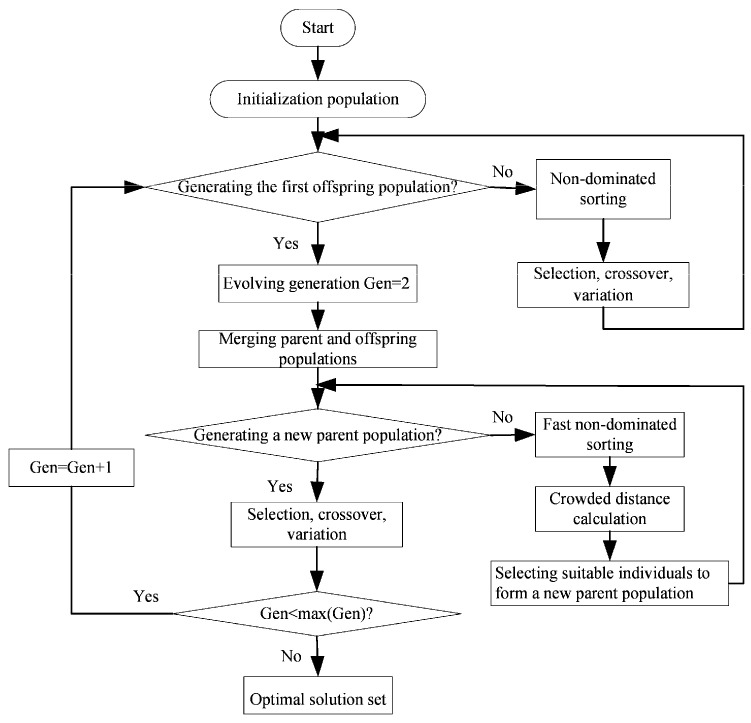
Flow chart of NSGA-II.

The evolution flow chart of NSGA-II is shown in Figure 10. The optimization results obtained by the combination of different objective functions in the three solutions are listed in Table 2. It can be seen that when single-objective optimization is performed under the criterions of maximum $\bar{P}$,η, $\bar{E}$, and ${\bar{P}}_{d}$, the deviation indexes (0.5828, 0.5210, 0.2086, and 0.4122, respectively) obtained are much larger than the result obtained by MOO. This indicates that the design scheme of MOO is more ideal. When taking $\bar{P}$, $\bar{E}$, and ${\bar{P}}_{d}$ as the optimization objectives to perform tri-objective optimization, the deviation index obtained by the LINMAP solution is smaller, and the design scheme is closer to the ideal scheme.

**Figure 11:**
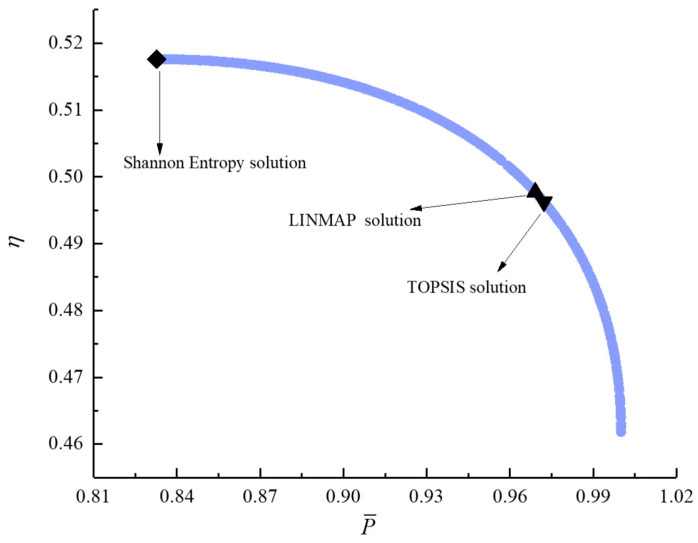
Bi-objective optimization on $\bar{P}-\eta$.

**Figure 12:**
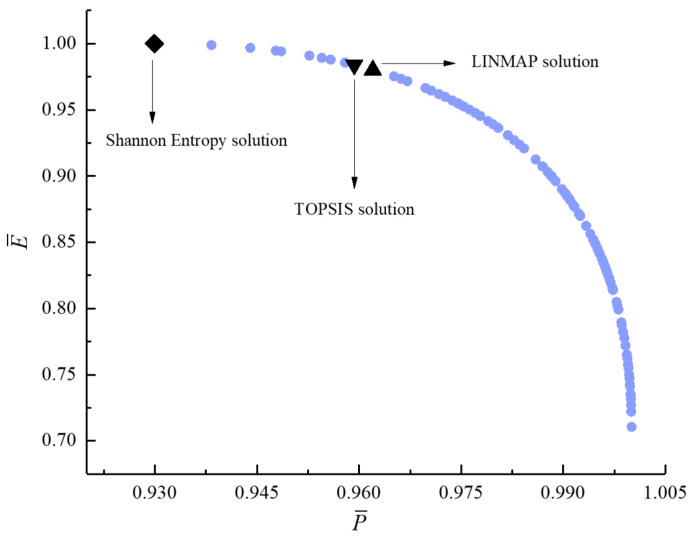
Bi-objective optimization on $\bar{P}-\bar{\underset{˙}{E}}$.

**Figure 13:**
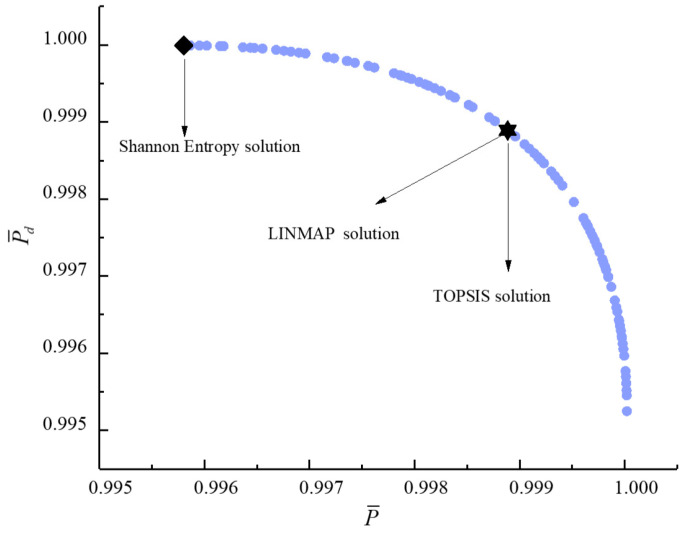
Bi-objective optimization on $\bar{P}-{\bar{P}}_{d}$.

**Figure 14:**
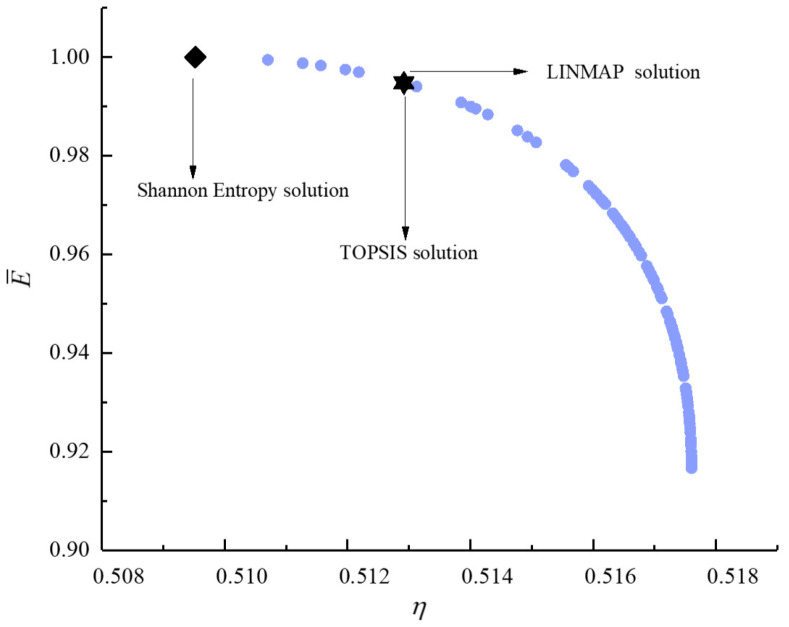
Bi-objective optimization on $\eta -\bar{\underset{˙}{E}}$.

**Figure 15:**
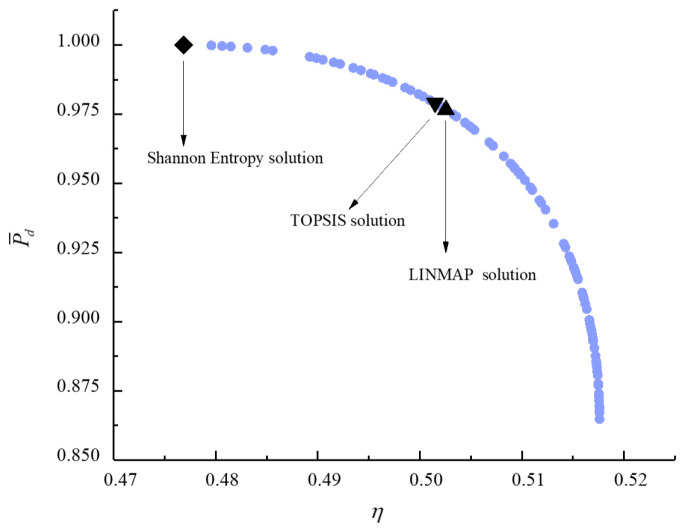
Bi-objective optimization on $\eta -{\bar{P}}_{d}$.

**Figure 16:**
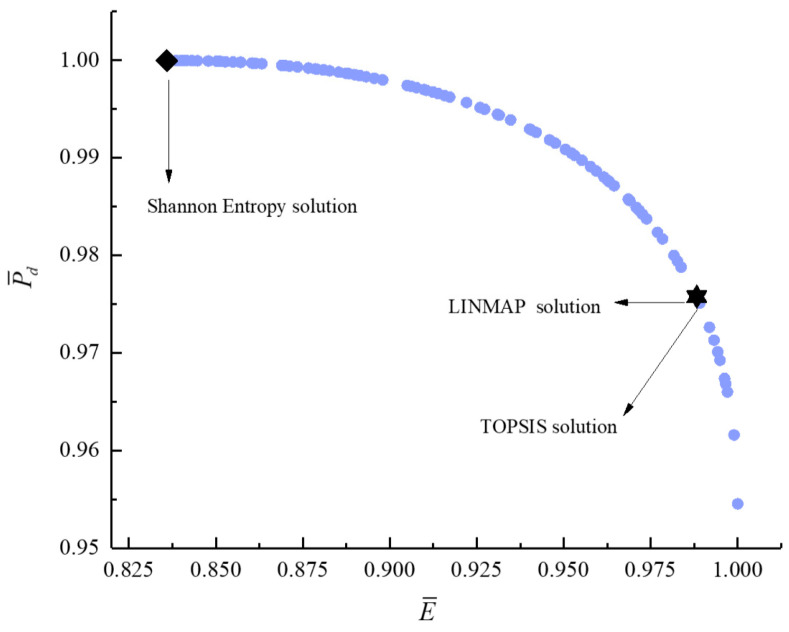
Bi-objective optimization on $\bar{E}-{\bar{P}}_{d}$.

Figure 11, Figure 12, Figure 13, Figure 14, Figure 15 and Figure 16 show the Pareto frontiers of bi-objective optimization ($\bar{P}-\eta$, $\bar{P}-\bar{\underset{˙}{E}}$, $\bar{P}-{\bar{P}}_{d}$, $\eta -\bar{\underset{˙}{E}}$, $\eta -{\bar{P}}_{d}$, and $\bar{E}-{\bar{P}}_{d}$). When $\bar{P}$ increases, η, $\bar{E}$, and ${\bar{P}}_{d}$ all decrease; when η increases, $\bar{E}$ and ${\bar{P}}_{d}$ both decrease; when $\bar{E}$ increases, ${\bar{P}}_{d}$ decreases. According to Table 1, when $\bar{P}$ and η or $\bar{P}$ and $\bar{E}$ are the objective functions, the deviation index obtained by the LINMAP solution is smaller. When $\bar{P}$ and ${\bar{P}}_{d}$ or η and $\bar{E}$ are the optimization objectives, the deviation index obtained by the Shannon entropy solution is smaller. When $\bar{E}$ and ${\bar{P}}_{d}$ are the optimization objectives, the deviation indexes obtained by the LINMAP and TOPSIS solutions are smaller than those obtained by the Shannon entropy solution. When η and ${\bar{P}}_{d}$ are the objective functions, the deviation index obtained by the TOPSIS solution is smaller.

**Figure 17:**
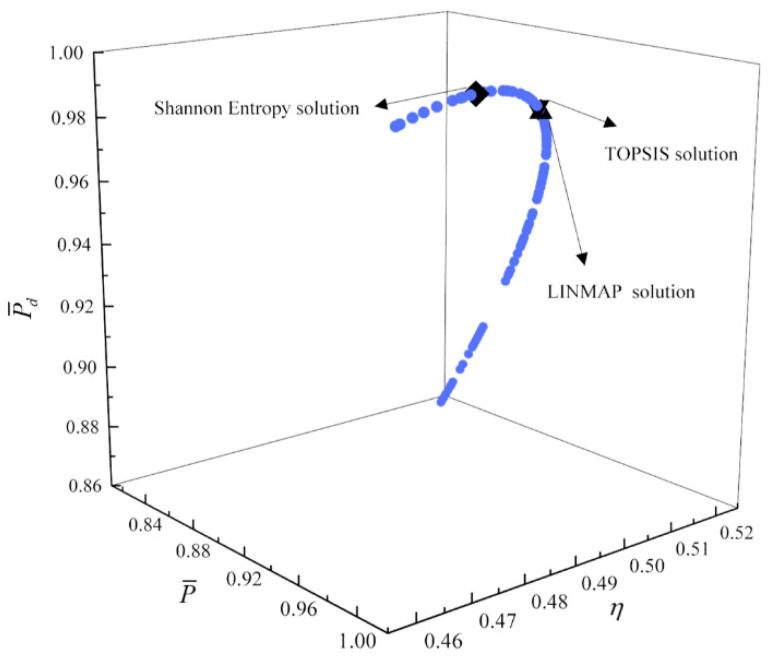
Tri-objective optimization on $\bar{P}-\eta -{\bar{P}}_{d}$.

**Figure 18:**
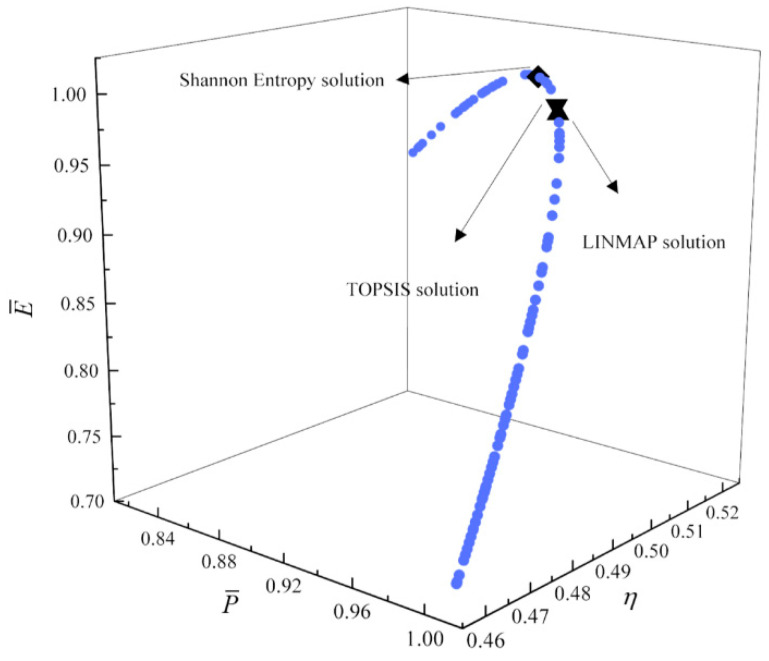
Tri-objective optimization on $\bar{P}-\eta -\bar{E}$.

**Figure 19:**
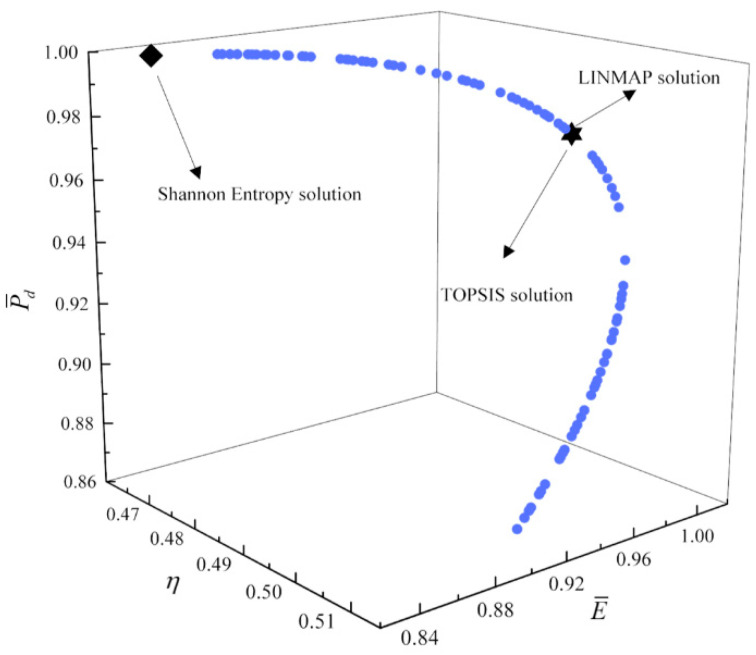
Tri-objective optimization on $\eta -\bar{E}-{\bar{P}}_{d}$.

**Figure 20:**
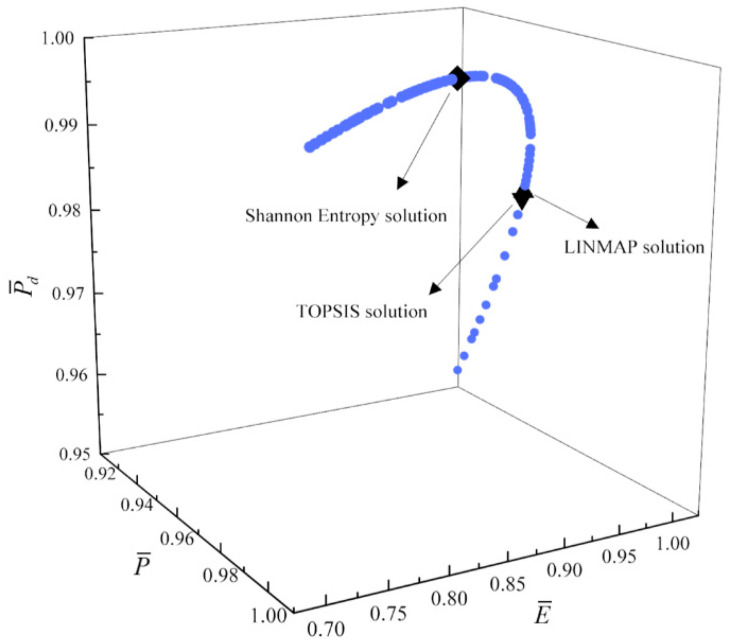
Tri-objective optimization on $\bar{P}-\bar{E}-{\bar{P}}_{d}$.

Figure 17, Figure 18, Figure 19 and Figure 20 show the Pareto frontiers of the tri-objective optimization ($\bar{P}-\eta -{\bar{P}}_{d}$, $\bar{P}-\eta -\bar{E}$, $\eta -\bar{E}-{\bar{P}}_{d}$, and $\bar{P}-\bar{E}-{\bar{P}}_{d}$). When $\bar{P}$ increases, η decreases, and $\bar{E}$ and ${\bar{P}}_{d}$ first increase and then decrease. When η increases, ${\bar{P}}_{d}$ decreases, and $\bar{E}$ first increases and then decreases. When η, $\bar{E}$, and ${\bar{P}}_{d}$ are the optimization objectives, the deviation indexes obtained by the LINMAP and TOPSIS solutions are smaller than those obtained by the Shannon entropy solution. When the combination of the other three objective functions are the optimization objectives, the deviation index obtained by the LINMAP solution is smaller, and the result is better.

**Figure 21:**
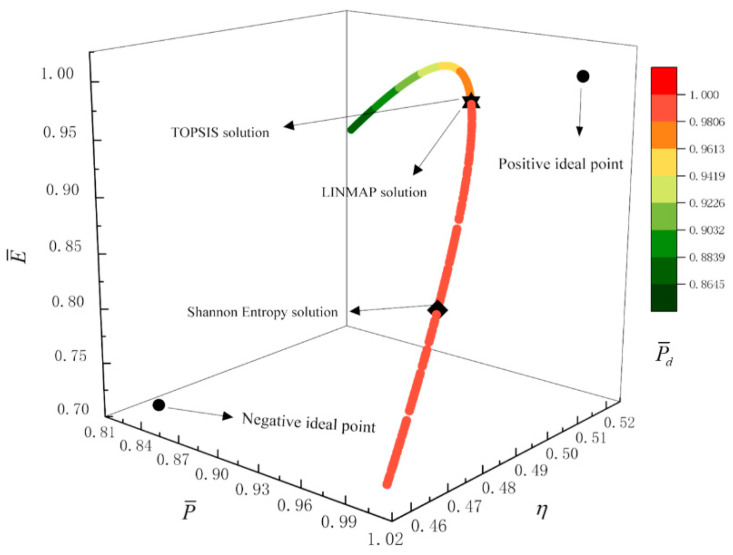
Quadru-objective optimization on $\bar{P}-\eta -\bar{E}-{\bar{P}}_{d}$.

Figure 21 shows the Pareto frontier of the quadru-objective optimization ($\bar{P}-\eta -\bar{E}-{\bar{P}}_{d}$). With the increase in $\bar{P}$, η increases, ${\bar{P}}_{d}$ decreases, and $\bar{E}$ first increases and then decreases. When $\bar{P}$, η, $\bar{E}$, and ${\bar{P}}_{d}$ are the optimization objectives, the deviation index obtained by the LINMAP solution is the smallest, and the result is the best.

## 5. Conclusions

The expression of the $P_{d}$ of an irreversible Diesel cycle was derived in this paper, and the impacts of τ and three loss issues on the cycle of $P_{d}$ versus γ and η characteristics were analyzed. The performance parameters (maximum specific volume, maximum pressure ratio, and η) of an irreversible Diesel cycle based on the criteria of maximum $\bar{P}$ and ${\bar{P}}_{d}$ were compared. Using three different solutions, including LINMAP, TOPSIS, and Shannon entropy, the results of single-, bi-, tri-, and quadru-objective optimization for an irreversible Diesel cycle were analyzed and compared. Comparing the deviation indexes obtained under different objective function combinations, the optimal design scheme was selected. The results showed the following:

1. The relationship curves of the cycles ${\bar{P}}_{d}-\gamma$ and ${\bar{P}}_{d}-\eta$ were a parabolic-like one and a loop-shaped one, respectively. With the increases in the cycle temperature ratio, the $\gamma_{{\bar{P}}_{d}}$ and $\eta_{{\bar{P}}_{d}}$ corresponding to the maximum ${\bar{P}}_{d}$ increased. With the increases in HFL, FL, and IIL, the $\gamma_{{\bar{P}}_{d}}$ and $\eta_{{\bar{P}}_{d}}$ corresponding to the maximum ${\bar{P}}_{d}$ decreased.
2. Under the maximum ${\bar{P}}_{d}$ criterion, a smaller size and higher efficiency engine will be designed.
3. The deviation index of MOO was smaller. When taking $\bar{P}$, $\bar{E}$, and ${\bar{P}}_{d}$ as the optimization objectives to perform tri-objective optimization, the deviation index obtained by the LINMAP solution was smaller, and the design scheme was closer to the ideal scheme.
4. The next step will be to use exergy efficiency optimization to further reinforce the results of MOO.

## Figures and Tables

**Table 1:** Comparison of the $\eta_{{\bar{P}}_{d}}$ in 8 cases.

Curve Number	Considered Loss	$\eta_{{\bar{P}}_{d}}$	$\mathrm{Percentage}\;\mathrm{of}\;\eta_{{\bar{P}}_{d}}\mathrm{Decrease}$
1	No loss	61.51%	0%
2	FL	60.36%	1.87%
3	HTL	56.45%	8.23%
4	FL and HTL	55.41%	9.92%
1′	IIL	52.97%	13.88%
2′	IIL and FL	51.84%	15.72%
3′	IIL and HTL	48.67%	20.87%
4′	IIL, HTL and FL	47.64%	22.55%

**Table 2:** Optimization results obtained by combining different objective functions.

Optimization Methods	Solutions	Optimization Variable	Optimization Objectives				Deviation Index
		γ	$\bar{P}$	η	$\bar{E}$	${\bar{P}}_{d}$	D
Quadru-objective optimization ($\bar{P}$, η, $\bar{E}$, and ${\bar{P}}_{d}$)	LINMAP	18.0466	0.9615	0.5008	0.9809	0.9804	0.1342
	TOPSIS	18.0822	0.9611	0.5010	0.9815	0.9801	0.1346
	Shannon entropy	14.3437	0.9958	0.4769	0.8359	1.0000	0.4068
Tri-objective optimization ($\bar{P}$, η, and $\bar{E}$)	LINMAP	18.2403	0.9591	0.5017	0.9842	0.9785	0.1366
	TOPSIS	18.5159	0.9556	0.5029	0.9882	0.9758	0.1422
	Shannon entropy	20.3584	0.9299	0.5095	1.0000	0.9545	0.2068
Tri-objective optimization ($\bar{P}$, η, and ${\bar{P}}_{d}$)	LINMAP	17.1965	0.9715	0.4966	0.9624	0.9878	0.1443
	TOPSIS	16.8933	0.9749	0.4949	0.9540	0.9900	0.1574
	Shannon entropy	14.3433	0.9958	0.4768	0.8359	1.0000	0.4068
Tri-objective optimization ($\bar{P}$, $\bar{E}$, and ${\bar{P}}_{d}$)	LINMAP	17.8459	0.9640	0.4999	0.9772	0.9823	0.1333
	TOPSIS	17.9598	0.9626	0.5004	0.9793	0.9812	0.1336
	Shannon entropy	14.3437	0.9958	0.4768	0.8359	1.0000	0.4068
Tri-objective optimization (η, $\bar{E}$, and ${\bar{P}}_{d}$)	LINMAP	18.7911	0.9520	0.5040	0.9916	0.9729	0.1495
	TOPSIS	18.7911	0.9520	0.5040	0.9916	0.9729	0.1495
	Shannon entropy	14.3437	0.9958	0.4769	0.8359	1.0000	0.4068
Bi-objective optimization ($\bar{P}$ and η)	LINMAP	17.4129	0.9691	0.4977	0.9678	0.9860	0.1380
	TOPSIS	17.3189	0.9722	0.4962	0.9655	0.9868	0.1384
	Shannon entropy	26.2726	0.8327	0.5176	0.9166	0.8647	0.5193
Bi-objective optimization ($\bar{P}$ and $\bar{E}$)	LINMAP	18.0043	0.9620	0.5006	0.9802	0.9808	0.1339
	TOPSIS	18.2236	0.9593	0.5016	0.9839	0.9787	0.1364
	Shannon entropy	20.3584	0.9299	0.5095	1.0000	0.9545	0.2068
Bi-objective optimization ($\bar{P}$ and ${\bar{P}}_{d}$)	LINMAP	13.5850	0.9989	0.4699	0.7800	0.9989	0.5004
	TOPSIS	13.5850	0.9989	0.4699	0.7800	0.9989	0.5004
	Shannon entropy	14.3437	0.9958	0.4768	0.8359	1.0000	0.4068
Bi-objective optimization (η and $\bar{E}$)	LINMAP	21.6879	0.9097	0.5129	0.9948	0.9367	0.2645
	TOPSIS	21.6879	0.9097	0.5129	0.9948	0.9367	0.2645
	Shannon entropy	20.3584	0.9299	0.5095	1.0000	0.9545	0.2068
Bi-objective optimization (η and ${\bar{P}}_{d}$)	LINMAP	18.4344	0.9566	0.5026	0.9871	0.9766	0.1403
	TOPSIS	18.1938	0.9597	0.5015	0.9834	0.9790	0.1359
	Shannon entropy	14.3437	0.9958	0.4768	0.8359	1.000	0.4068
Bi-objective optimization ($\bar{E}$ and ${\bar{P}}_{d}$)	LINMAP	18.5178	0.9555	0.5029	0.9882	0.9758	0.1422
	TOPSIS	18.5178	0.9555	0.5029	0.9882	0.9758	0.1422
	Shannon entropy	14.3437	0.9958	0.4769	0.8359	0.9999	0.4068
Maximum of $\bar{P}$	-	12.8106	1.0000	0.4617	0.7090	0.9952	0.5828
Maximum of η	-	26.2980	0.8323	0.5176	0.9160	0.8643	0.5210
Maximum of $\bar{E}$	-	20.4061	0.9293	0.5096	1.0000	0.9540	0.2086
Maximum of ${\bar{P}}_{d}$	-	14.3205	0.9960	0.4765	0.8330	1.0000	0.4122
Positive ideal point		-	1.0000	0.5176	1.0000	1.0000	-
Negative ideal point		-	0.8328	0.4618	0.7105	0.8647	-

## Data Availability

Data sharing not applicable.

## Nomenclature

B	Heat transfer loss coefficient (W/K)
$C_{p}$	Specific heat at constant pressure (J/(mol⋅K))
$C_{v}$	Specific heat at constant volume (J/(mol⋅K))
$\bar{E}$	Dimensionless ecological function
$\bar{P}$	Dimensionless power output
${\bar{P}}_{d}$	Dimensionless power density
Q	Heat transfer rate (W)
*T*	Temperature (K)
Greek symbols	
γ	Compression ratio (-)
η	Thermal efficiency (-)
μ	Friction coefficient (kg/s)
σ	Entropy generation rate (W/K)
τ	Temperature ratio (-)
Subscripts	
${\bar{P}}_{d}$	Max power density condition
0	Environment
1 − 4,2s,4s	Cycle state points
Abbreviations	
FL	Friction loss
HTL	Heat transfer loss
IIL	Internal irreversibility loss
MOO	Multi-objective optimization
